# Changes in Adherence to Non-Pharmacological Guidelines for Hypertension

**DOI:** 10.1371/journal.pone.0161712

**Published:** 2016-08-25

**Authors:** Kyong Park, Sukyung Cho, Julie K. Bower

**Affiliations:** 1 Department of Food and Nutrition, Yeungnam University, Gyeongsan, Gyeongbuk, Republic of Korea; 2 Division of Epidemiology, The Ohio State University College of Public Health, Columbus, Ohio, United States of America; Shanghai Institute of Hypertension, CHINA

## Abstract

This study aimed to compare levels of adherence to non-pharmacological guidelines between patients with and without hypertension diagnoses, and examined temporal changes in adherence during recent decades. We used data from the Korean National Health and Nutrition Examination Survey (1998–2012), including 13,768 Korean hypertensive patients aged ≥ 30 years who were categorized according to the presence or absence of a hypertension diagnosis, based on blood pressure and self-reported information. Adherence to the guidelines was calculated for 6 components, including dietary and lifestyle habits. A multivariable generalized linear regression model was used. The proportion of hypertensive patients aware of their condition increased from 33.4% in 1998 to 74.8% in 2012 (*p* < 0.001), although these increments plateaued during recent survey years. Patients with hypertension diagnoses were older, and more likely to be female,and have lower education levels than those without hypertension diagnoses, for most survey years. Overall adherence levels were poor (mean score 2 of 6), and levels of adherence to non-pharmacological habits did not significantly differ between patients with and without hypertension diagnoses. However, overall adherence levels improved significantly among patients with hypertension diagnoses: from 2.09 in 1998 to 2.27 in 2012 (*p* = 0.007), particularly regarding sufficient vegetable/seaweed consumption (*p* = 0.03), maintaining a normal weight (*p* = 0.03), and avoidance of smoking (*p* < 0.001). Awareness of hypertension is increasing, but hypertensive Korean patients demonstrate poor overall adherence to non-pharmacological hypertension management guidelines. These findings suggest that well-planned education programs should be continued after hypertension is diagnosed.

## Introduction

Although hypertension has a high prevalence in Asian populations, its management and patient adherence to lifestyle recommendations have not been a priority [1]. According to the Korea National Health and Nutrition Examination Surveys (KNHANES), the prevalence of hypertension has stabilized in recent decades, at approximately 30% for Korean adults aged ≥ 30 years [2]. High blood pressure is often asymptomatic, but long-term hypertension increases the risk of serious complications, such as coronary heart disease [3, 4]. These complications account for 9.4 million deaths every year [5], and hypertension is a considerable public health problem worldwide [6].

Hypertension is both preventable and manageable by modifying diet and lifestyle [6], as indicated by established clinical guidelines [7]. These modifications are generally termed “non-pharmacological modifications”, and are characterized by the high intake of fresh fruits and vegetables, moderate intake of low-fat dairy and healthy proteins (poultry, fish, and nuts), low intake of sodium, weight management, and regular physical activity [8]. There has been great interest in non-pharmacological approaches in recent decades, as such approaches are safer and can enhance the effectiveness of antihypertensive drug therapy, compared to drugs alone [9]. Cumulative evidence also suggests that greater adherence to a healthy diet and lifestyle has been associated with lower blood pressure [10] and a decreased risk of chronic disease [11] and mortality [12].

Although better adherence to guidelines is associated with enhanced health benefits through improved blood pressure control, limited information is available regarding the population-level adherence to these guidelines among hypertensive Korean patients. In Korea, the guidelines for management of hypertension were primarily developed by the Ministry of Health and Welfare (MHW) [13] and the Korean Society of Hypertension [14]. These 2 sets of guidelines are similar in that they advocate a low-sodium diet, and a higher intake of fiber-rich fruits and vegetables to maintain a healthy weight.

In 2001, the MHW established the National Hypertension Center, a national organization that links researchers and health care providers to develop effective strategies for preventing hypertension in South Korea. The aim of the National Hypertension Center was to decrease the prevalence of hypertension and related complications, and increase opportunities for education on hypertension as a means of facilitating self-management, through a variety of national interventions, particularly via establishment of a community-based program at public health centers. After 2007, case management programs for hypertensive patients were implemented, and consisted of visiting and interviewing by trained health professionals. Several prior reports have examined the effects of this case management program. So et al. [15] reported that a 8-12-week case management program resulted in significant improvements in blood pressure, body weight loss, salt and vegetable intake (lower sodium and higher vegetable intake), and physical activity in 30 participants with hypertension. Similarly, Chung et al. [16] demonstrated the efficacy of this case management program in controlling blood pressure. However, these studies were conducted with small sample sizes and were geographically limited; thus, the results could not be generalized. In addition, limited information is available regarding whether disease awareness is associated with greater levels of adherence to the guidelines, and how adherence levels have changed over time in the Korean population. Therefore, we compared levels of adherence to the non-pharmacological guidelines between patients with and without hypertension, and examined changes in adherence levels between the 1998 and 2012 surveys, using the KNHANES data.

## Materials and Methods

### 2.1 Population

We analyzed data from the KNHANES 1998–2012, a nationally representative cross-sectional survey that used a complex, stratified, multistage, probability-cluster sampling approach. The protocols and measurements of the KNHANES have been described in detail elsewhere [17]. Briefly, the surveys collect extensive information through a health interview, health examination, and nutritional survey. The KNHANES was conducted as a series of 3 surveys in 1998, 2001, and 2005 for 10 weeks. Since 2007, the KNHANES have been performed year-round to avoid seasonal variations affecting data.

All participants signed an informed consent form, and the study was approved by the Institutional Review Board (IRB) of the Korea Centers for Disease Control and Prevention, who also provided formal ethics approval for the KNHANES data sets (IRB numbers: 2007-02-CON-04-P, 2008-04EXP-01-C, 2009-01CON-03-2C, 2010-02CON-21-C, 2011-02CON-06-C, 2012-01EXP-01-2C).

Adults at or above 30 years of age tend to exhibit changes in metabolic processes with age [18–21]; such changes increase the importance of lifestyle adherence to hypertension management [22, 23]. Consequently, this analysis included participants greater than or equal to 30 years of age. Among a total 41,586 participants aged 30 years or older and without missing information on blood pressure, sampling weight, and dietary intake, we excluded individuals who had implausible energy intakes (< 500 kcal/day or > 5000 kcal/day) (n = 567), and non-hypertensive subjects (n = 27,251). We only included patients with hypertension if they met at least 1 of the following conditions: (1) measured systolic blood pressure (SBP) ≥ 140 mmHg or diastolic blood pressure (DBP) ≥ 90 mmHg [7], (2) use of antihypertensive medication, (3) a medical history of hypertension as diagnosed by a physician, or (4) self-reported hypertension. Among defined hypertensive patients, those who gave positive responses to questions (2), (3), or (4) were defined as having diagnosed hypertension, and the remainder were considered to have undiagnosed hypertension. A total of 13,768 hypertensive participants were included in the final analysis.

### 2.2 Measurements

Demographic and lifestyle characteristics including age, sex, education, household income, smoking, alcohol consumption, and physical activity were collected through a health interview questionnaire. Smoking status was categorized as never, former, or current. Alcohol intake was calculated by multiplying the servings of alcohol consumed in 1 sitting by the frequency of consumption. Physical activity information could not be obtained for the 1998 and 2001 surveys, and some details were omitted from the 2005 and 2007 surveys. Thus, physical activity variables were used to calculate metabolic equivalents (METs [h/week]) [24] from the 2008–2012 surveys only.

Dietary information was obtained through a single 24-hour recall method, and the participants were asked to recall every consumed food item, as well as meal times, eating places, and the quantity of food consumed (volume [ml] or weight [g]). All consumed foods were coded and transformed to nutrients, including carbohydrate and sodium, on the basis of the Food Composition Table by the Rural Development Administration [25]. Consumption levels of vegetable and seaweed were examined using individual intake data from the 24-hour dietary recall, and each item was calculated with the exception of kimchi and pickled vegetables. Serving sizes of food items were defined according to the Dietary Reference Intakes for Koreans [26].

Anthropometric measurements were standardized and performed by trained members of the survey team. Height in centimeters was measured to the nearest 0.1 cm with a wall-mounted stadiometer (Seriter: Seca, Bismarck, ND, USA in 1998; Seriter Stadiometer: Holtain Ltd; Crosswell, UK in 2001; Seca model 225: Hamburg, Germany in 2005–2012), and weight in kilograms was determined to 0.1 kg using an digital scale (Giant-150N: Hana, Seoul, Korea in 1998, DA150W: Dana, Incheon, Korea in 2001; GL-6000-20: CAS, Seoul, Korea in 2005–2012). Body mass index (BMI) was calculated as weight divided by height squared (weight [kg]/height [m^2^]), and “normal weight” was defined based on the report from the World Health Organization for Asian populations. BMI values falling into the range 18.5 kg/m^2^ ≤ BMI < 23 kg/m^2^ were considered normal weight, and those where the BMI was ≥ 23 kg/m^2^ were considered overweight/obese [27]. Waist circumference was measured as the abdominal girth midway between the costal margin and the iliac crest (Seca model 220; Seca, Hamburg, Germany).

Blood pressure was measured by trained medical staff using a mercury sphygmomanometer (Baumanometer; Baum, USA). In 1998 and 2001, blood pressure measurements were taken twice in a stable state, and the mean SBP and DBP values were used, while 3 repeated measurements were averaged for the analysis after 2005. From 2011, the KNHANES conducted a project to evaluate the quality of blood pressure measurements and to provide prospects for quality improvement [28].

### 2.3 Non-pharmacological guidelines for hypertensive patients

Assessment of level of adherence to non-pharmacological guidelines was determined on the basis of the “Dietary Guidelines for Disease Management” [13] developed by the MHW, and the “Korean Hypertension Treatment Guidelines” [29] by the Korean Society of Hypertension. The level of adherence to non-pharmacological recommendations was calculated based on 6 components, as follows (Box 1):

**Box 1. Nutritional and lifestyle components of non-pharmacological recommendations**

[Nutritional components]

1. Limited sodium intake (< 2000 mg/day)
2. Sufficient intakes of fresh vegetables and seaweeds (≥ 6~7 servings per day, except kimchi and pickles)
3. Moderate carbohydrate consumption (≤ 60% of total energy intake) [Lifestyle components]
4. Moderate alcohol consumption (≤ 2 cups per day for men and ≤ 1 cup per day for women)
5. Maintaining a normal weight (18.5 kg/m^2^ ≤ BMI < 23 kg/m^2^)
6. No current smoking

Individuals were assigned a value of 1 if they adhered to each component or 0 if they did not, with the total possible score for overall adherence ranging from 0 (the lowest adherence) to 6 (the highest adherence).

### 2.4 Statistical analysis

All statistical analyses were conducted with consideration of the complex survey design that used a multistage, stratified, clustered sampling approach to obtain a study sample representative of the entire non-institutionalized South Korean population. Descriptive statistics of participants were obtained by determining weighted frequency distributions of categorical variables and weighted means ± standard error of continuous variables. Characteristics of patients with and without hypertension diagnoses were compared using the chi-square test for categorical variables or the t-test for continuous variables. Multivariable generalized linear regression analysis was performed using the SAS PROC SURVEYREG command, which provides means and 95% confidence intervals (CI), to give estimates of adherence levels for diagnosed and undiagnosed hypertension. *P*-values were based on 2-sided tests, and significance was determined at α = 0.05. Statistical analyses were performed using SAS V9.2 (SAS Institute Inc., Cary, NC, USA).

## Results

Demographic and lifestyle information for Korean hypertensive patients is summarized in Table 1. The estimated proportion of persons with hypertension increased from 31.2% in 1998 to 39.4% in 2012. The mean age of hypertensive patients increased by approximately 7.6 years during the 14 years of the survey period (*p* for trend < 0.001). One-third of hypertensive patients graduated high school or higher, and two-thirds of them were obese (BMI ≥ 23 kg/m^2^). Smoking rates significantly decreased from 34.8% in 1998 to 14.6% in 2012 (*p* for trend < 0.001), while a significant decrease was also observed for physical activity levels (*p* for trend < 0.001) during the recent survey years of 2008 to 2012.

**Table 1:** Characteristics of adults aged ≥30 years with hypertension by survey year (N = 13,768).

	Survey year									*P* for trend ^1)^
	1998	2001	2005	2007	2008	2009	2010	2011	2012	
**N (%)**	1629(31.2)	1234(29.9)	1219(29.5)	742(31.7)	1689(32.7)	1924(33.9)	1676(34.5)	1830(37.6)	1825(39.4)	
**Age (years)** ^3)^	56.3±0.3	57.7±0.4	58.6±0.4	63.0±0.5	62.0±0.3	62.9±0.3	62.8±0.3	63.6±0.3	63.9±0.3	<0.001^2)^
**Male (%)** ^3)^	799(49.1)	600(48.6)	591(48.5)	326(43.9)	708(41.9)	889(46.2)	745(44.5)	813(44.4)	801(43.9)	0.1
**Overweight or obesity (%)**^3)^ ^4)^	1060(65.1)	871(70.6)	876(72.5)	545(74.2)	1191(71.6)	1347(70.2)	1161(69.5)	1254(68.8)	1266(69.4)	<0.001
**High school or higher (%)** ^3)^	525(32.2)	478(38.9)	456(37.9)	216(29.9)	535(31.9)	647(34.1)	590(35.9)	658(37.1)	652(38.4)	<0.001
**Household income (%)** ^3)^ ^5)^										
** Low**	409(25.1)	305(26.3)	349(28.8)	199(28.5)	397(24.5)	475(25.0)	429(25.9)	439(24.2)	454(25.3)	0.1
** Mid-low**	426(26.2)	273(23.6)	316(26.1)	167(23.9)	431(26.6)	499(26.3)	413(24.9)	501(27.7)	478(26.7)	
** Mid-high**	395(24.3)	322(27.8)	270(22.3)	164(23.5)	399(24.7)	452(23.8)	430(26.0)	463(25.6)	449(25.1)	
** High**	399(24.5)	259(22.4)	276(22.8)	169(24.2)	391(24.2)	473(24.9)	384(23.2)	408(22.5)	411(22.9)	
**Current smoker (%)** ^3)^	566(34.8)	369(29.9)	271(22.2)	125(17.2)	299(17.8)	312(16.3)	259(15.7)	295(16.6)	248(14.6)	<0.001
**Current alcohol drinker (%)** ^3)^	776(47.6)	661(54.1)	662(54.3)	424(58.4)	942(56.1)	1061(55.3)	908(55.5)	975(55.0)	942(55.5)	<0.001
**METs peer week (%)** ^3)^										
** <20**					759(45.4)	895(47.0)	863(52.6)	1022(57.8)	1027(60.8)	<0.001
** 20–39**					323(19.3)	343(18.0)	284(17.3)	316(17.9)	292(17.3)	
** ≥40**					589(35.3)	667(35.0)	493(30.1)	429(24.3)	369(21.9)	

^1)^ *P*-values were derived from a chi-square test for categorical variables.

^2)^ *P* for trend was derived from a linear regression analysis.

^3)^ Values are unweighted n (weighted %) or weighted mean ± standard error.

^4)^ Body Mass Index (kg/m^2^) ≥ 23.

^5)^ Household income quartiles were calculated based on equivalised income (total household income divided by the square root of the numbers of household members).

**Fig 1:**
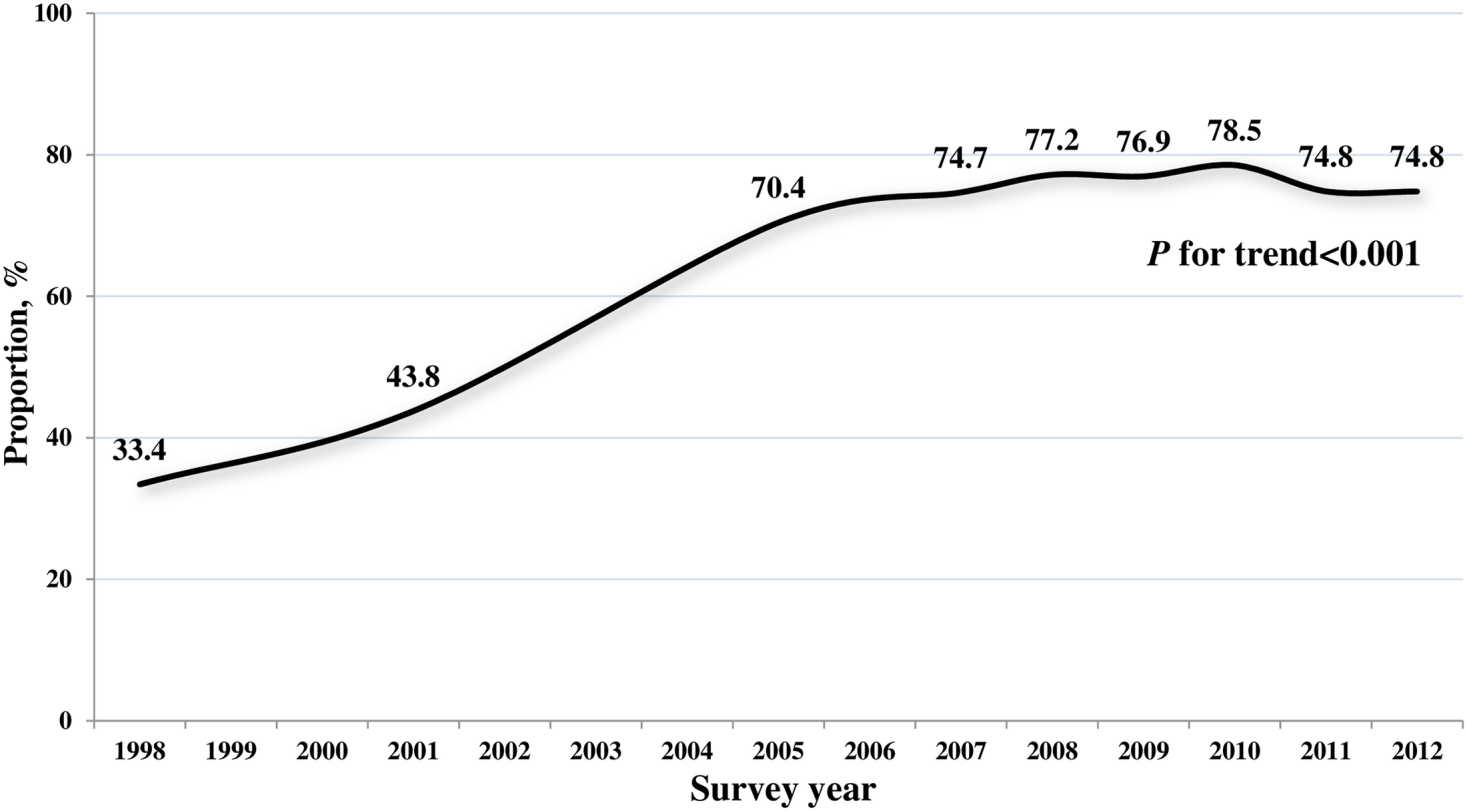
Trends in diagnosed hypertension (weighted proportion) among persons aged 30 years or older with self-reported hypertension or blood pressure ≥ 140/90 mmHg: KNHANES 1998–2012.

Among hypertensive patients, the proportion of diagnosed cases was 33.4% in 1998. This substantially increased until 2010, then plateaued in the 2011–2012 survey years (*p* for trend < 0.001, Fig 1).

Table 2 shows the general characteristics of hypertensive patients according to diagnosis status and survey year. Patients with hypertension diagnoses were older (*p* < 0.001) and were more likely to be female (*p* < 0.001), than those without; these differences were observed for all survey years. In addition, the group with hypertension diagnoses was more likely to have greater waist circumference, lower levels of education, and lower prevalence of current smoking than the group without hypertension diagnoses, for most survey years.

**Table 2:** Characteristics of hypertensive patients by awareness status (diagnosed/undiagnosed) and survey years (N = 13,768) ^1)^.

	**1998**			**2001**			**2005**		
	**Diagnosed**	**Undiagnosed**	***P*-value**	**Diagnosed**	**Undiagnosed**	***P*-value**	**Diagnosed**	**Undiagnosed**	***P*-value**
**N (%)**	544(33.4)	1085(66.6)		540(43.8)	694(56.2)		858(70.4)	361(29.6)	
**Age (years)**	58.0±0.7	53.2±0.6	<0.001	60.8±0.6	52.4±0.7	<0.001	60.0±0.5	51.1±0.9	<0.001^2)^
**Male (%)**	217(44.1)	582(57.6)	<0.001	210(42.5)	390(61.0)	<0.001	370(46.7)	221(67.4)	<0.001
**Overweight or obesity (%)**^2)^	396(74.0)	664(63.3)	<0.001	399(72.6)	472(67.9)	0.1	610(73.2)	266(74.2)	0.8
**High school or higher (%)**	137(30.8)	388(43.6)	<0.001	158(31.3)	320(51.6)	<0.001	282(37.0)	174(53.3)	<0.001
**Household income (%)**^3)^									
** Low**	141(23.3)	268(22.2)	0.07	129(24.8)	176(25.1)	0.8	239(24.1)	110(30.4)	0.06
** Mid-low**	125(21.6)	301(27.8)		124(25.6)	149(23.1)		235(29.3)	81(21.3)	
** Mid-high**	128(23.3)	267(23.4)		136(27.5)	186(30.0)		188(23.4)	82(25.1)	
** High**	150(31.9)	249(26.7)		117(22.1)	142(21.9)		190(23.2)	86(23.2)	
**Current smoker (%)**	152(30.0)	414(40.7)	<0.001	119(25.4)	250(39.1)	<0.001	164(20.6)	107(33.1)	<0.001
**Current alcohol drinker (%)**	311(57.2)	465(42.9)	<0.001	344(64.3)	317(46.1)	<0.001	514(59.9)	148(41.0)	<0.001
**METs per week (%)**									
** <20**									
** 20–39**									
** ≥40**									
	**2007**			**2008**			**2009**		
	**Diagnosed**	**Undiagnosed**	***P*-value**	**Diagnosed**	**Undiagnosed**	***P*-value**	**Diagnosed**	**Undiagnosed**	***P*-value**
**N (%)**	554(74.7)	188(25.3)		1303(77.2)	386(22.9)		1480(76.9)	444(23.1)	
**Age (years)**	61.0±0.8	55.2±1.5	<0.001	60.5±0.5	52.1±0.9	<0.001	61.8±0.4	54.2±0.9	<0.001
**Male (%)**	216(44.1)	110(62.6)	<0.001	522(45.9)	186(58.9)	<0.001	650(48.6)	239(61.8)	<0.001
**Overweight or obesity (%)**^2)^	409(77.5)	136(76.8)	0.9	950(76.1)	241(68.4)	0.01	1054(73.8)	293(68.1)	0.05
**High school or higher (%)**	151(35.9)	65(43.9)	0.2	376(36.5)	159(53.8)	<0.001	466(39.2)	181(51.5)	<0.001
**Household income (%)**^3)^									
** Low**	141(25.7)	58(30.4)	0.6	290(22.8)	107(27.7)	0.4	363(23.4)	112(25.1)	0.2
** Mid-low**	120(23.8)	47(24.9)		336(26.3)	95(24.8)		375(25.5)	124(29.1)	
** Mid-high**	130(23.7)	34(22.3)		315(24.8)	84(24.2)		348(23.5)	104(23.7)	
** High**	128(26.9)	41(22.3)		306(26.0)	85(23.2)		373(27.6)	100(22.1)	
**Current smoker (%)**	83(19.4)	42(25.7)	0.2	216(19.0)	83(28.8)	0.001	210(16.5)	102(29.5)	<0.001
**Current alcohol drinker (%)**	350(64.2)	74(40.9)	<0.001	773(59.7)	169(44.2)	<0.001	871(58.9)	190(43.0)	<0.001
**METs per week (%)**									
** <20**				589(44.7)	170(44.8)	0.9	705(45.9)	190(41.6)	0.006
** 20–39**				245(20.4)	78(21.2)		281(19.1)	62(14.8)	
** ≥40**				459(34.8)	130(34.0)		483(34.9)	184(43.6)	
	**2010**			**2011**			**2012**		
	**Diagnosed**	**Undiagnosed**	***P*-value**	**Diagnosed**	**Undiagnosed**	***P*-value**	**Diagnosed**	**Undiagnosed**	***P*-value**
**N (%)**	1316(78.5)	360(21.5)		1369(74.8)	461(25.2)		1365(74.8)	460(25.2)	
**Age (years)**	61.9±0.5	55.0±0.8	<0.001	63.5±0.5	52.1±0.9	<0.001	63.0±0.5	52.4±0.7	<0.001
**Male (%)**	557(44.0)	188(60.2)	<0.001	566(44.2)	247(64.2)	<0.001	550(44.7)	251(62.2)	<0.001
**Overweight or obesity (%)**^2)^	912(72.1)	249(71.0)	0.7	961(72.0)	293(70.7)	0.7	954(69.0)	312(69.7)	0.8
**High school or higher (%)**	424(34.9)	166(55.3)	<0.001	435(34.1)	223(58.7)	<0.001	429(38.4)	223(58.9)	<0.001
**Household income (%)**^3)^									
** Low**	331(26.7)	98(27.8)	0.9	324(25.5)	115(27.2)	0.8	323(26.0)	131(30.5)	0.3
** Mid-low**	315(25.1)	98(25.9)		375(28.6)	126(29.7)		366(28.2)	112(24.7)	
** Mid-high**	347(25.5)	83(24.9)		348(24.9)	115(24.1)		347(25.5)	102(23.2)	
** High**	309(22.8)	75(21.4)		308(21.0)	100(19.0)		303(20.4)	108(21.7)	
**Current smoker (%)**	181(17.8)	78(29.6)	<0.001	186(17.4)	109(32.1)	<0.001	144(14.1)	104(30.2)	<0.001
**Current alcohol drinker (%)**	754(58.6)	154(44.0)	<0.001	788(59.3)	187(42.2)	<0.001	758(60.0)	184(42.3)	<0.001
**METs per week (%)**									
** <20**	697(53.3)	166(46.7)	0.03	804(61.9)	218(45.8)	<0.001	772(61.9)	255(58.0)	0.5
** 20–39**	225(17.4)	59(14.5)		220(16.8)	96(21.9)		216(17.7)	76(18.4)	
** ≥40**	368(29.2)	125(38.8)		300(21.3)	129(32.3)		267(20.4)	102(23.6)	

^1)^ Values are unweighted n (weighted %) or weighted mean ± standard error.

^2)^ Body Mass Index (kg/m^2^) ≥ 23.

^3)^ Household income quartiles were calculated based on equivalised income (total household income divided by the square root of the numbers of household members).

**Fig 2:**
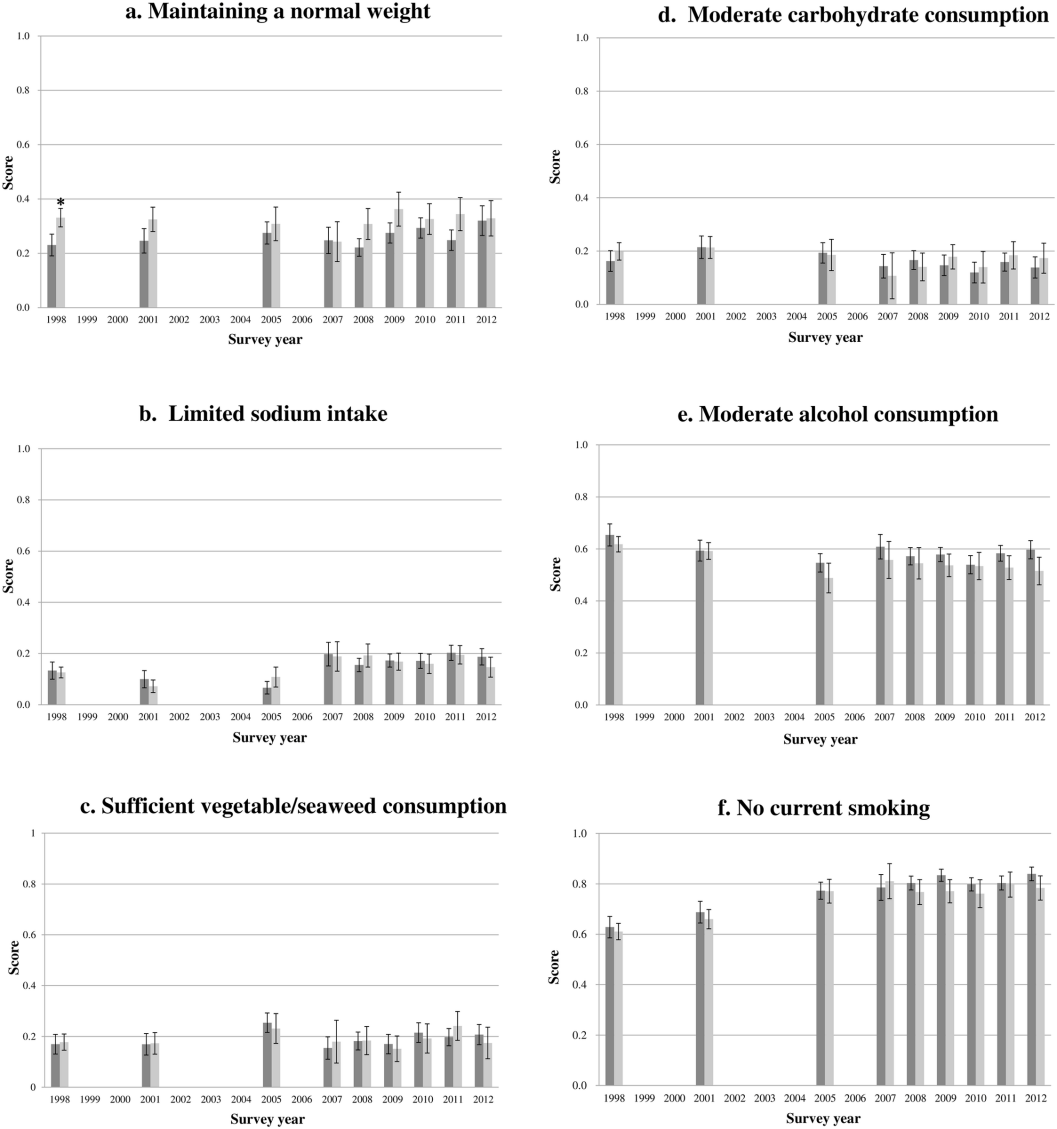
Mean adherence levels and 95% confidence intervals for each recommendation, after adjusting for age, sex, education, household income, alcohol consumption (except for e), and smoking status (except for f) during 1998–2012. Each point ranges from 0 (non-adherence) to 1 (perfect adherence); dark grey bars are for patients with hypertension diagnoses (HD) and light grey bars are for those without diagnoses (UD). Multivariable general linear models were used to test differences in levels of adherence to each recommendation between the HD and UD patients for all survey years. * Adjusted means are significantly different at *p* < 0.05.

Levels of adherence to the 6 non-pharmacological guidelines among patients with and without hypertension diagnoses during the 1998–2012 survey years were examined in a multivariable linear regression after adjusting for age, sex, education, household income, alcohol consumption, and smoking status (Fig 2). Adherence to recommended non-pharmacological habits did not significantly differ according to diagnosis status for most survey years, with the exception of maintaining a normal weight. Patients without hypertension diagnoses had a higher level of adherence to maintaining a healthy weight (BMI from 18.5 to 23 kg/m^2^) than patients with hypertension diagnoses; however, such an observation was only made in 1998 (undiagnosed: 0.33 points vs diagnosed: 0.23 points). The guidelines with the greatest adherence ratings were avoidance of smoking (0.61–0.84 points) and moderate alcohol consumption (0.49–0.65 points). However, nutrient-related guidelines, such as limiting sodium intake (0.07–0.20 points), moderate carbohydrate consumption (0.10–0.21 points), and sufficient intakes of fresh vegetables and seaweeds (0.15–0.24 points) exhibited poor adherence.

**Fig 3:**
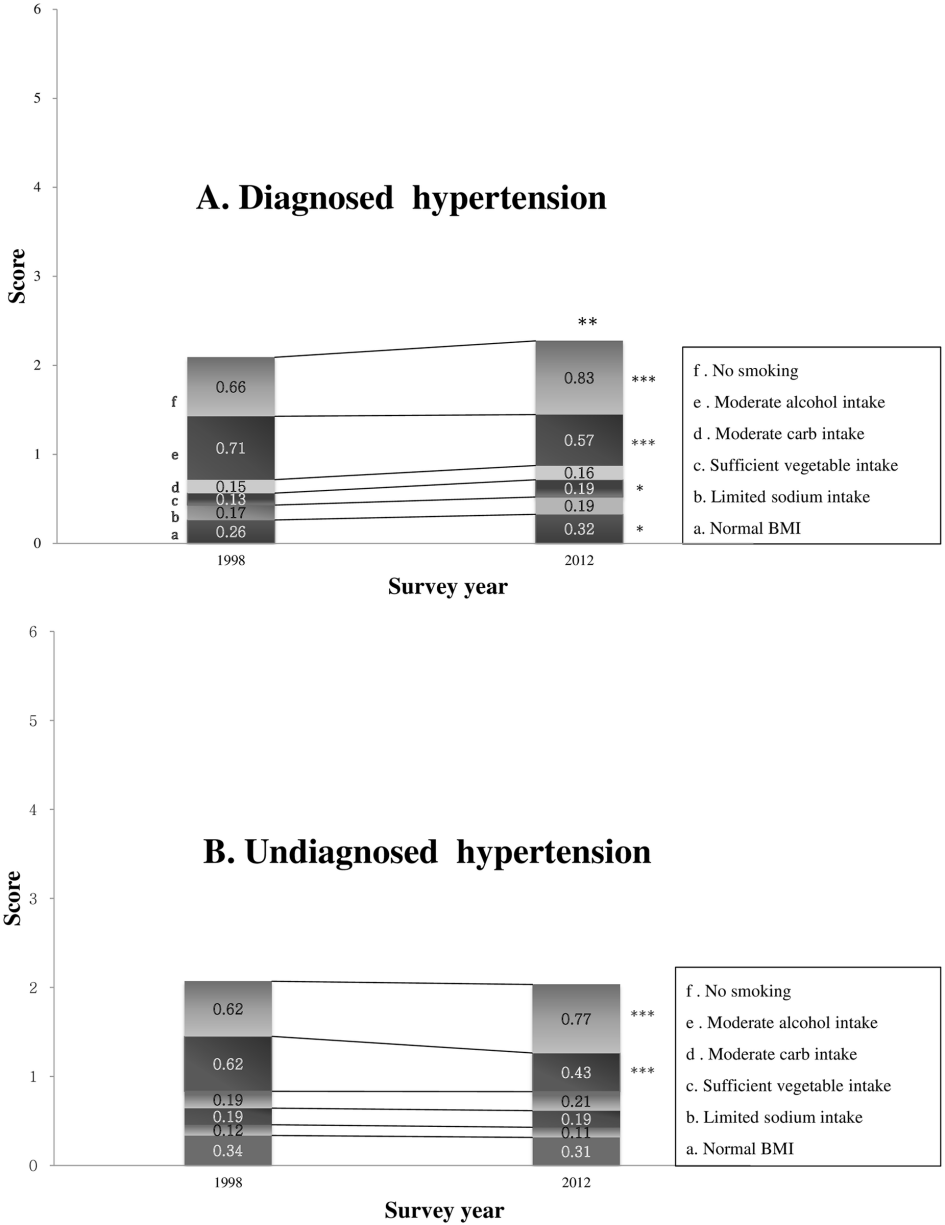
Changes in overall mean adherence levels between patients with and without hypertension diagnoses from 1998 to 2012. Estimates were adjusted for age, sex, education, and household income. * Adjusted means are significantly different at *p* < 0.05; ** adjusted means are significantly different at *p* < 0.01; *** Adjusted means are significantly different at *p* < 0.001.

Fig 3 shows changes in the levels of adherence to the 6 non-pharmacological guidelines as well as overall adherence among patients with and without hypertension diagnoses, between 1998 and 2012. There were significant changes in levels of adherence to no current smoking and moderate alcohol consumption for patients with and with hypertension diagnoses. Levels of adherence to no current smoking increased from 0.66 in 1998 to 0.83 in 2012 among diagnosed patients (*p* < 0.001), and from 0.62 in 1998 to 0.77 in 2012 among undiagnosed patients (*p* < 0.001). However, adherence to moderate alcohol consumption decreased from 0.71 in 1998 to 0.57 in 2012 among diagnosed patients (*p* < 0.001), and from 0.62 in 1998 to 0.43 in 2012 among undiagnosed patients (*p* < 0.001). Adherence to sufficient vegetable/seaweed consumption (*p* = 0.03) and maintaining a normal weight (*p* = 0.03) improved over time for diagnosed subjects only. Overall adherence to the 6 non-pharmacological guidelines ranged between 2.09–2.27 and 2.07–2.03 among patients with and without hypertension diagnoses, respectively; however, improvement was significant in diagnosed patients only (*p* = 0.007).

**Fig 4:**
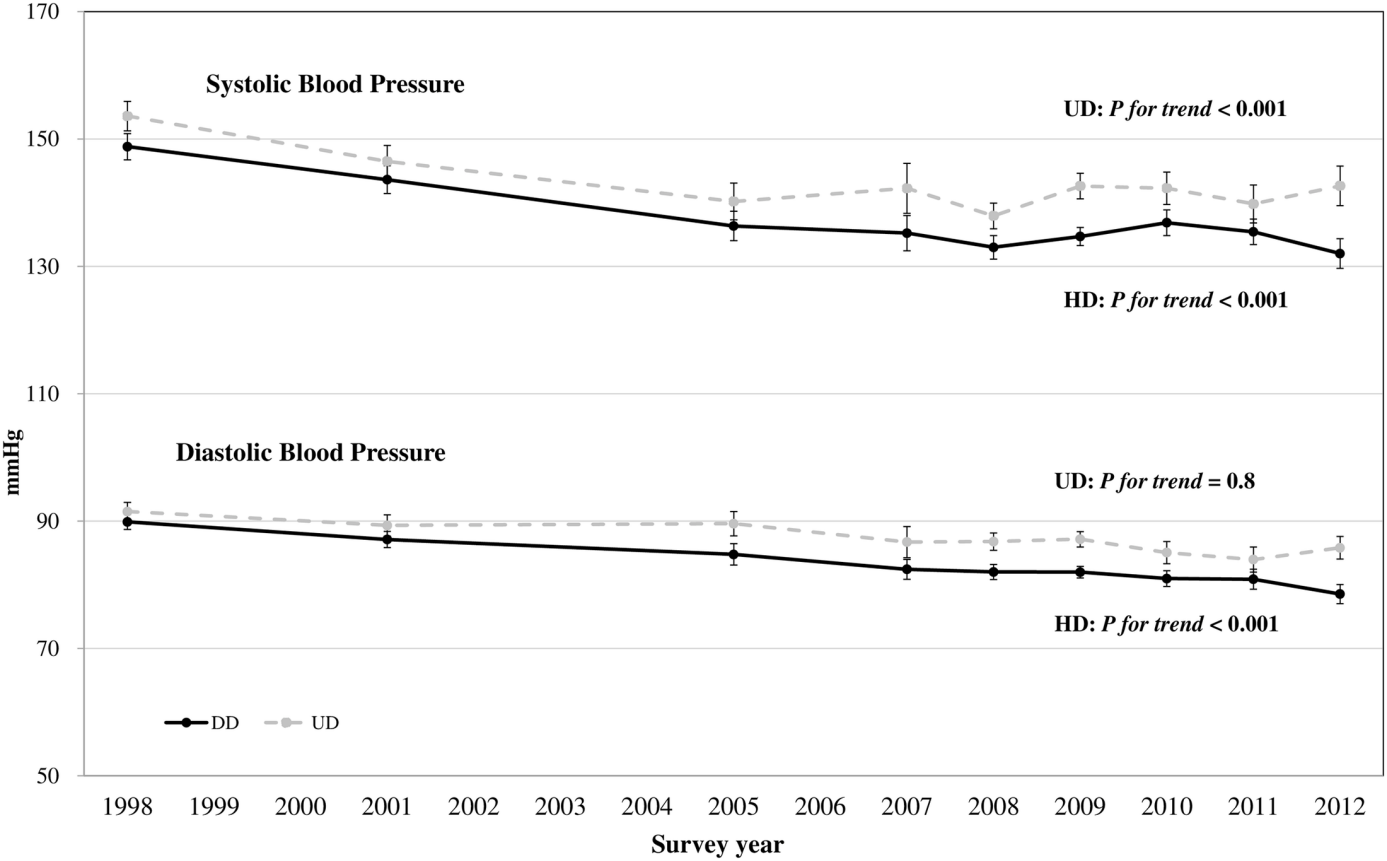
Trends in mean systolic and diastolic blood pressures and 95% confidence intervals for patients with and without hypertension diagnoses from 1998 to 2012. Estimates were adjusted for age, sex, and use of antihypertensive agents. Solid lines are for patients with hypertension diagnoses (HD), and dotted lines are for those without diagnoses (UD).

Fig 4 illustrates the trends in average SBP and DBP, adjusted for age, sex, and the use of antihypertensive agents, for patients with and without hypertension diagnoses. From 1998 to 2012, the average levels of SBP significantly decreased from 148.79 mmHg in 1998 to 132.01 mmHg in 2012 among diagnosed patients (*p* for trend < 0.001), and from 153.59 mmHg in 1998 to 142.64 mmHg in 2012 among undiagnosed patients (*p* for trend < 0.001). The average levels of DBP decreased by 11.33 mmHg during 14 years for diagnosed patients (*p* for trend < 0.001), but no significant change in DBP was observed for undiagnosed patients (decreased by 5.8 mmHg, *p* for trend = 0.8). Overall, both SBP and DBP tended to be higher in undiagnosed patients than diagnosed patients for most survey years.

## Discussion

During recent decades, the proportion of patients diagnosed with hypertension has rapidly increased. However, overall levels of adherence to non-pharmacological guidelines for blood pressure management have been poor, and a hypertension diagnosis was not associated with better adherence to the guidelines when compared to persons with undiagnosed hypertension, for most survey years. Overall adherence levels improved slightly among patients with hypertension diagnoses. However, this was not observed among patients with undiagnosed hypertension between the 1998–2012 survey years. Smoking rates were relatively low and decreased substantially over time for both patients with and without hypertension diagnoses. Improvements in adherence may be responsible for positive reductions in blood pressure, particularly for patients with hypertension diagnoses whose blood pressure has significantly decreased over the 14 survey years.

Our results demonstrate that awareness of hypertension has improved over the past decade in Korea, and remained stable in 2010–2012, with a high proportion of the hypertensive population reporting a diagnosis (74.5–78.5%). According to cumulative scientific reports, similar awareness levels have been reported in the US, however, relatively low levels of awareness have been observed in China. A National Health and Nutrition Examination Survey (NCHS) data brief [29] reported that awareness levels were high in the US, with approximately 80.2% of male and 85.4% of female hypertensive patients reporting awareness of their condition. Interestingly, Asian adults with hypertension were less aware of their condition (72.8%) than other races, such as non-Hispanic white (82.7%), non-Hispanic black (85.7%), and Hispanic (82.2%) individuals. According to a recent report regarding Chinese hypertension guidelines, the prevalence of hypertension has increased, but the awareness of hypertension was only 30.2% among Chinese hypertensive adults aged 18 years or older [30].

In addition, hypertension awareness was associated with other factors. For example, patients who were diagnosed were older [31, 32], more likely to be female [33], had lower rates of smoking [32], reported a family history of hypertension [31], had a higher household income [31], and a higher BMI [32] than those with undiagnosed hypertension.

Patient awareness of hypertension is important for achieving blood pressure control, because it provides opportunities to change diet and lifestyle habits, and initiate pharmacological therapy where warranted. In the European Investigation into Cancer and Nutrition-NL cohort, subjects aware of their hypertension were more likely to adhere to the guidelines, particularly for fat and fiber intake, alcohol restriction, and achievement and maintenance of a healthy weight [34]. However, we found no differences in adherence to the non-pharmacological guidelines between patients with and without hypertension diagnoses. Plausible explanations exist for these discrepant results. For example, a lack of motivation may be a root cause of low adherence among hypertensive patients to pursue dietary and lifestyle changes. Hypertension has been labeled “the silent killer” because it has no early symptoms; thus, hypertensive patients may be less motivated to change their dietary and lifestyle until they experience serious health conditions, such as a stroke or heart attack [35]. Therefore, it is particularly important to emphasize the impact of changes in dietary and lifestyle habits through focused education programs targeting Korean patients after a diagnosis of hypertension.

One of the least followed guidelines was limiting sodium intake, which is particularly important for hypertensive patients. High sodium intake is known to increase sympathetic nervous system activity and cardiac output, and eventually increases the risk of hypertension and its complications such as cardiovascular disease [36]. Observational studies and randomized controlled trials have repeatedly shown that higher dietary sodium intake is associated with higher blood pressures [37, 38] and an increased risk of hypertensive complications [39] and mortality [40]. The average sodium intake for Korean adults is 4,878 mg/day [2], more than 2.4 times the recommended amount [41], because the traditional Korean diet includes various high-sodium dishes. The major sources of sodium are kimchi (1125 mg, 24.5%), noodles (572 mg, 12.4%), soups (488 mg, 10.6%), and stews (399 mg, 8.7%) [42]. In addition, salt sensitivity of blood pressure is present in approximately half of the Korean population with essential hypertension [43], which is attributable to an impaired renal capacity to excrete sodium [44]. This predisposition corresponds to an increase in health risks associated with cardiovascular disease [45]. Therefore, Korean patients with hypertension must closely monitor the sodium content in their diet.

Although the overall levels of adherence to non-pharmacological guidelines were poor, and did not differ between patients with and without hypertension diagnoses for all survey years, there were significant positive changes in adherence levels among patients with hypertension diagnoses in recent decades. These improvements were apparent in adherence to avoiding smoking, sufficient vegetable/seaweed consumption, and maintenance of a normal weight. This may be a positive sign of government effort (e.g., conducting and expanding community-based intervention programs at public health centers nationally) to improve patient self-management skills for hypertension. In fact, in this population, we observed a trend towards decreasing blood pressure levels, particularly for patients with hypertension diagnoses. Focused interest and investment in education programs for hypertensive patients will be necessary at the population level, and improvements in levels of adherence to non-pharmacological guidelines for hypertension will be key to reducing the public health burden of complications secondary to hypertension.

Our study has several strengths. By obtaining a large population and representative data from the KNHANES, our results are more generalizable to the South Korean hypertensive population. In addition, to our knowledge, this may be the first study to examine levels of adherence to non-pharmacological guidelines, considering diagnosis status as well as changes over time.

Limitations of this study should also be considered. Although blood pressure was measured by trained medical staff and the average used to identify diagnosed and undiagnosed hypertension, misclassification may exist, as identification of hypertension was based on self-reported information, whereby confounders, such as the “white coat” phenomenon, may change the observed results. In addition, this was a cross-sectional study and therefore could not examine changes over time within individual. Dietary and nutrient intake levels were evaluated using a 1-day 24-h recall method, which could have resulted in misclassification. Finally, our participants were recruited from 1998 to 2012, and our definition of non-pharmacological guidelines was primarily based on 2 guidelines, the “Dietary Guidelines for Disease Management” (2010) and the “2013 Korean Hypertension Treatment Guidelines” (2013). The first edition of the Korean Hypertension Treatment Guidelines (2004) was not considered in our definition, but was almost identical to the 2013 Korean Hypertension Treatment Guidelines with respect to non-pharmacological components.

In conclusion, awareness of hypertension may be important for patients to control blood pressure. However, awareness itself may be insufficient to motivate patients to change their dietary and lifestyle behaviors. Thus, it is critical to improve patient education efforts to better manage hypertension and improve compliance with dietary and lifestyle recommendations, such that blood pressure can be controlled aggressively to prevent long-term complications.
